# Histopathology and quantification of green fluorescent protein-tagged *Fusarium oxysporum* f. sp. *luffae* isolate in resistant and susceptible *Luffa* germplasm

**DOI:** 10.1128/spectrum.03127-23

**Published:** 2024-01-04

**Authors:** Ahmed Namisy, Shu-Yun Chen, Jin-Hsing Huang, Jintana Unartngam, Chinnapan Thanarut, Wen-Hsin Chung

**Affiliations:** 1 Department of Plant Pathology, National Chung Hsing University, Taichung, Taiwan; 2 Department of Agronomy, National Chung Hsing University, Taichung, Taiwan; 3 Plant Pathology Division, Taiwan Agricultural Research Institute, Council of Agriculture, Taichung, Taiwan; 4 Department of Plant Pathology, Faculty of Agriculture, Kasetsart University, Bangkok, Thailand; 5 Faculty of Agriculture Production, Division of Pomology Maejo University, Bangkok, Thailand; 6 Innovation and Development Center of Sustainable Agriculture (IDCSA), National Chung Hsing University, Taichung, Taiwan; 7 Master Program for Plant Medicine and Agricultural Practice, National Chung Hsing University, Taichung, Taiwan; Universita degli Studi del Molise, Campobasso, Italy

**Keywords:** *Fusarium oxysporum* f. sp.* luffae*, GFP, disease resistance, *Luffa acutangula*

## Abstract

*Fusarium oxysporum* f. sp. *luffae* (Folu) is a severe plant pathogen that causes vascular wilt and root rot in *Luffa* plants worldwide. A green fluorescent protein (GFP)-tagged isolate of Folu (Fomh16-GFP) was utilized to investigate the infection progress and colonization of Fomh16-GFP in resistant (LA140) and susceptible (LA100) *Luffa* genotypes. Seven days post-inoculation (dpi), it was observed that Fomh16-GFP had successfully invaded and colonized the vascular bundle of all LA100 parts, including the roots, hypocotyl, and stem. Pathogen colonization continued to increase over time, leading to the complete wilting of plants by 14–17 dpi. In LA140, the Fomh16-GFP isolate colonized the roots and hypocotyl vascular system at 7 dpi. Nevertheless, this colonization was restricted in the hypocotyl and decreased significantly, and no fungal growth was detected in the vascular system at 21 dpi. Thus, the resistant genotype might trigger a robust defense mechanism. In addition, while the pathogen was present in LA140, the inoculated plants did not exhibit any symptoms until 28 dpi. Quantitative PCR was utilized to measure the Fomh16-GFP biomass in various parts of LA100 and LA140 at different time points. The findings indicated a positive correlation between the quantity of Fomh16-GFP DNA and disease development in LA100. Alternatively, a high amount of Fomh16-GFP DNA was identified in the roots of LA140. Nonetheless, no significant correlations were found between DNA amount and disease progression in LA140. Aqueous extracts from LA140 significantly reduced Fomh16-GFP spore germination, while no significant reduction was detected using LA100 extracts.

**IMPORTANCE:**

Fusarium wilt of *Luffa*, caused by *Fusarium oxysporum* f. sp. *luffae* (Folu), causes great losses in *Luffa* plants worldwide. This study used a green fluorescent protein (GFP)-tagged isolate of Folu (Fomh16-GFP) to investigate the infection progress and colonization dynamics of Fomh16-GFP in the resistant and susceptible *Luffa* genotypes, which could be important in understanding the resistance mechanism of Folu in *Luffa* plants. In addition, our work highlights the correlations between DNA amount and disease progression in resistant plants using real-time PCR. We observed a positive correlation between the quantity of Fomh16-GFP DNA and disease progression in LA100, while no significant correlation was found in LA140. These results could be valuable to further investigate the resistance mechanism of *Luffa* genotypes against Folu. Gaining a better understanding of the interaction between Folu and *Luffa* plants is crucial for effectively managing Fusarium wilt and enhancing resistance in *Luffa* rootstock and its varieties.

## INTRODUCTION

*Luffa* spp. are a significant vegetable among cucurbits in tropical and subtropical countries (1). The two cultivated *Luffa* species, Ridge gourd [*Luffa acutangula* (L.) Roxb.] and sponge gourd [*Luffa cylindrica* (L.) Roem.], are widely grown in Asia (2). *Luffa* plants are an adequate source of nutrients for humans, and immature fruits are consumed as vegetables in Asia and Africa (3). Along with their nutritional benefits, *Luffa* plants are a standard rootstock for bitter gourd (*Momordica charantia*) in Taiwan. It is widely used for summer cultivation because of its resistance to Fusarium wilt and tolerance to heat and flooding (4). Several diseases have been reported in *Luffa*, including Fusarium wilt caused by *Fusarium oxysporum* f. sp. *luffae* (Folu).

*Fusarium oxysporum* (Fo) is a severe plant pathog en that causes vascular wilt and root rot in a diverse group of plants (5). The pathogen is distributed worldwide in soil and occurs in the rhizosphere (6, 7). With a wide range of hosts, Fo can cause devastating vascular wilt in more than 100 plant species (8). Currently, the pathogen ranks fifth among the top 10 fungal plant pathogens (9). Fusarium wilt in *Luffa*, caused by *Fusarium oxysporum* f. sp. *luffae*, causes great losses in *Luffa* plant yields worldwide. The pathogen penetrates the roots and colonizes the vascular tissue of susceptible plants. The initial symptoms appear as stunted growth, yellowing, wilting, and, finally, plant death (10, 11), while the internal symptoms include brown discoloration in vascular tissue, which is visible in stem cross-sections (12). Several methods have been used to manage *F. oxysporum*, including chemical, physical, and biological methods (13). However, these methods only partially control Fusarium wilt because the pathogen can survive for long periods in the soil as chlamydospores which can colonize the residues and roots of most crops (7, 14).

Additionally, no efficient treatments exist to cure a plant after its infection, and growers generally have to remove them from their crops (15). Cultivars with resistance are the most effective defense against soil-borne diseases. Fusarium wilt-resistant cultivars have been reported in *L. acutangula* and *L. aegyptiaca* (16). However, the resistance mechanism against Folu in the *Luffa* species is largely unknown.

GFP is a valuable marker for studying plant–microbe interactions, including those with fungi and bacteria (17, 18). The processes of infection and colonization by Fo in the susceptible and resistant genotypes have been investigated using GFP-tagged Fo isolates in cucurbit crops such as watermelon (19), cucumber (5, 20), and melon (21). The relationship between colonization and vascular wilt of several plant species has been reported in watermelon seedlings infected with *F. oxysporum* f. sp. *niveum* (Fon) race 1 (22). More research studies have indicated that Fon could penetrate and colonize the roots of the highly resistant watermelon line but failed to enter the vascular system (23) and did not result in wilt symptoms (24). A previous study reported that four Folu isolates colonized the hypocotyl of highly resistant *Luffa* accessions without displaying symptoms (16). This behavior might be similar to Fon in that Folu colonizes hypocotyl in the resistant *Luffa* but does not move into the vascular system.

Conventional PCR assays are practical, specific, and highly sensitive for identifying *F. oxysporum* (25). It can detect the *Fusarium* species early, even before the symptoms occur on the plant (26). This method’s limitation is that it cannot quantify the *Fusarium* species in soil and plant tissues (27). In contrast, the application of quantitative PCR (qPCR) might provide the ability to detect, quantify, and distribute a specific pathogen in the host plant (28), which could be used to investigate the infection epidemiology and plant–pathogen interactions (29). Identifying and quantifying the pathogen in the early stages of infection can assist in managing the pathogen and reduce crop losses (30). The qPCR assay has been developed to identify and quantify the amount of Fon in watermelon plant tissues and soil (27), *F. oxysporum* f. sp. *melonis* (Fom) in melon cultivars (31), and *F. oxysporum* f. sp. *cucumerinum* (Foc) in cucumber root tissue and soil (29). A high-sensitivity qPCR protocol was developed to detect and quantify Fom in resistant and susceptible melon stems. This allows for detection in the early infection stage, even without displaying any symptoms (31). The purpose of this research study was twofold: (i) to examine the disease progression and colonization of GFP-tagged Folu Fomh16-GFP isolate in the susceptible and resistant *Luffa* genotypes and (ii) to investigate the correlation between the quantity of Fomh16-GFP DNA and disease development in the susceptible and resistant *Luffa* genotypes.

## RESULTS

### Generation and characterization of the transformed Fomh16-GFP isolate

The growth of wild-type Fomh16 isolate was inhibited when grown in PDA media containing 100 µg/mL hygromycin B. We used 200 µg/mL of hygromycin B in the selective media. The transformed colonies were grown on nitrocellulose membrane filters for 7 dpi on selective media at 25°C. To determine the stability or instability of the transformants, independent transformant colonies were sequentially transferred five times to a fresh selective medium. Only the transformants that grew well were selected, and a single spore was cultured on a non-selective PDA medium at 25°C for 2 weeks. The representative Fomh16-GFP isolate was characterized further.

**Fig 1:**
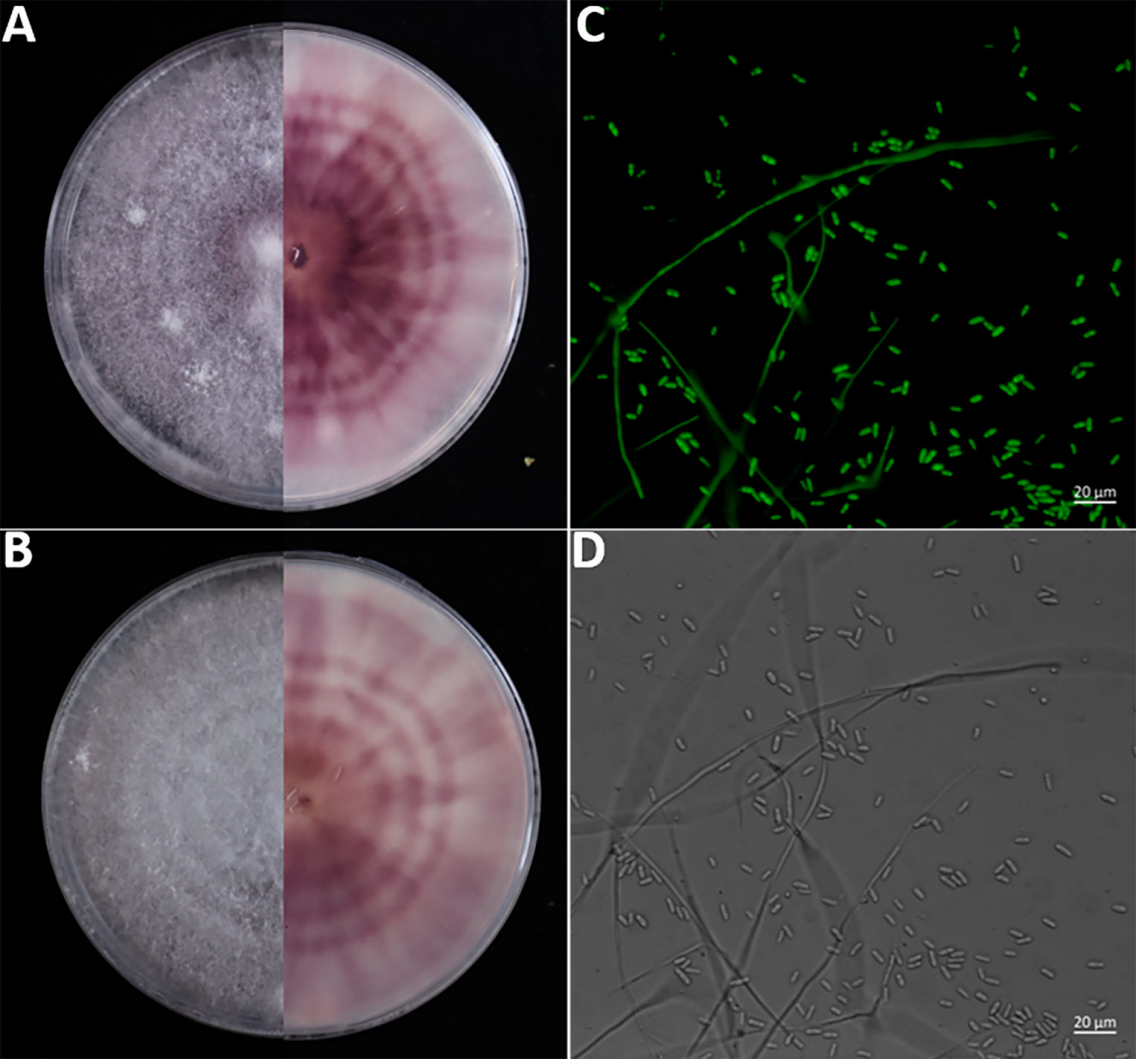
Phenotypic characterization of the wild-type Fomh16 and transformed Fomh16-GFP isolates. (A) Forward and reverse of colony morphology of Fomh16-GFP; (B) forward and reverse of colony morphology of Fomh16; (C) uniform expression of GFP in mycelium and microconidia of Fomh16-GFP; (D) mycelium and microconidia of Fomh16-GFP under a light microscope.

**Fig 2:**
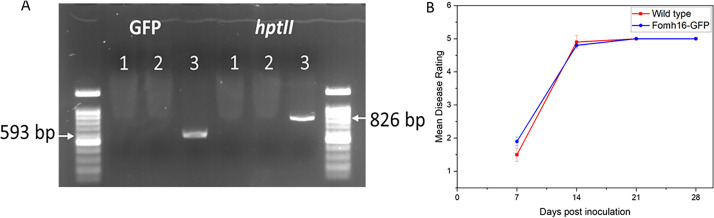
Molecular characterization and pathogenicity tests of the transformed isolate. (A) PCR-amplified specific fragments of the *hptII* and GFP genes in Fomh16-GFP and Fomh16 isolates, 1, non-template DNA; 2, Fomh16 isolate; 3, Fomh16-GFP isolate; (B) pathogenicity test of both isolates against susceptible *Luffa* genotype LA100.

The transformed Fomh16-GFP isolates exhibited similar morphological characteristics to wild-type Fomh16 (Fig. 1). Both isolates produced cottony light purple aerial mycelium, with time purple pigmentation appearing. Only microconidia with an ovoid shape were observed on PDA, and no significant differences in mycelial growth rate and spore germination percentage were observed with either isolate (data not shown). The presence of the GFP and *hptII* genes in the transformed isolates was confirmed using PCR with specific primers (GFP_F and GFP_R) and (HygR3 and HygR5), respectively. Both genes were amplified from the transformed isolate, showing the expected sizes: 826 bp for *hptII* and 593 bp for GFP. Neither gene could be amplified in the wild-type Fomh16 isolate or in the non-template control (Fig. 2A). The pathogenicity of wild-type Fomh16 and transformed Fomh16-GFP was tested against a highly susceptible *Luffa* genotype LA100. These results indicated that both isolates were highly pathogenic to *Luffa* plants with a mean disease rating (MDR) of 5 at 21 dpi (Fig. 2B).

### Colonization of the Fomh16-GFP isolate in *Luffa* genotypes

The resistant genotype LA140 and susceptible genotype LA100, with one to two true leaves, were inoculated with *F. oxysporum* f. sp. *luffae* Fomh16-GFP. The LA100 genotype started displaying symptoms at 5 dpi. Initially, the symptoms included wilting with an MDR of 1.5 at 7 dpi and 100% plant disease incidence. Moreover, the wilting progressed rapidly, and by 14 to 20 dpi, most plants had completely wilted with an MDR of 5. In contrast with genotype LA100, the resistant genotype LA140 showed a highly resistant reaction and displayed no symptoms at 28 dpi. As expected, no symptoms were observed in non-inoculated plants at 28 dpi.

**Fig 3:**
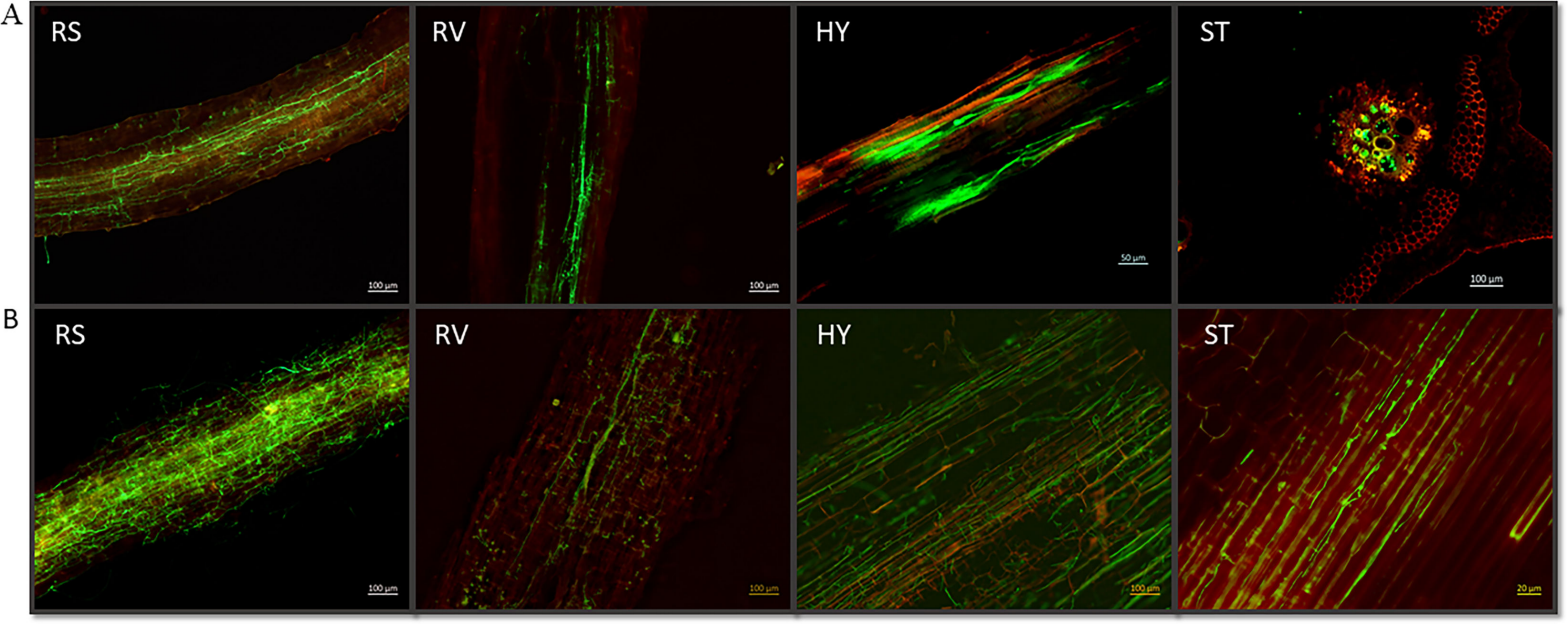
Infection and colonization of Folu Fomh16-GFP isolate in the susceptible *Luffa* genotype LA100. (A) Cross and longitudinal sections in the susceptible genotype at 7 dpi; (B) longitudinal sections in the susceptible genotype at 14 dpi. RS, root surface; RV, root vascular bundle; HY, hypocotyl; ST, stem.

We monitored the progression of pathogen colonization in two *Luffa* genotypes, LA100 (susceptible) and LA140 (resistant), after they were infected with the Fomh16-GFP isolate. The roots, hypocotyl, and stem sections were examined at different times. The susceptible *Luffa* genotype, LA100, was monitored for the colonization of the pathogen only at 7 and 14 dpi due to most of the plants completely wilting by 14 to 17 dpi. At 7 dpi, the lower and middle sections of the root surface showed significant mycelial colonization in the susceptible genotype (Fig. 3A). The mycelium penetrated the primary roots and colonized the xylem vessels. Additionally, observations revealed the presence of mycelium in the root cortex tissue. After 14 dpi, the mycelial colonization had expanded and covered nearly the entire root surface and vascular bundle (Fig. 3B). The LA100 genotype showed a low production of secondary roots at 7 dpi, and by 14 dpi, the pathogen had infected all roots, leading to their death and decay.

In addition, the mycelium showed extensive growth in the hypocotyl and stem and heavily colonized the vascular bundle at 7 dpi (Fig. 3A). Over time, pathogen colonization increased, and the mycelium spread throughout all hypocotyl and stem tissues at 14 dpi (Fig. 3B). Moreover, the microconidia and chlamydospores were developed in the root surface and by the root, hypocotyl, and stem vascular bundle at 7 and 14 dpi in LA100.

**Fig 4:**
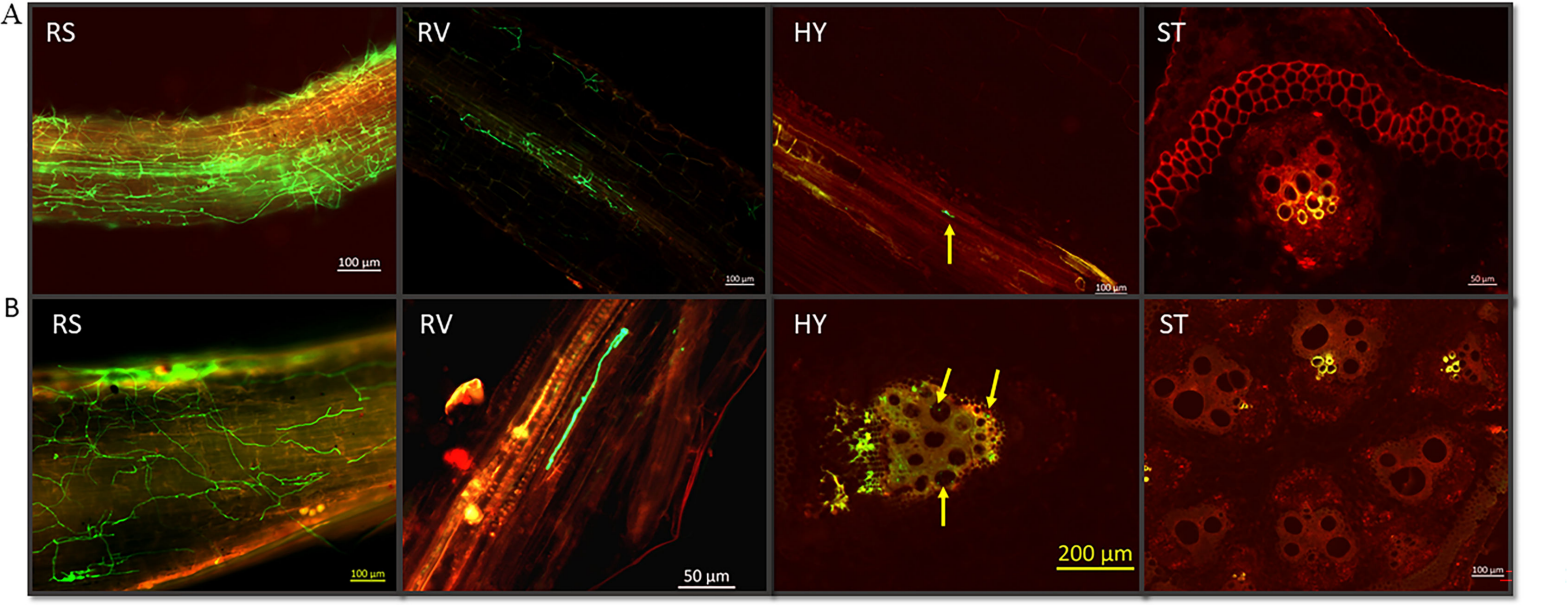
Infection and colonization of Folu Fomh16-GFP isolate in the resistant *Luffa* genotype LA140. (A) Cross and longitudinal sections in the resistant genotype at 7 dpi; (B) cross and longitudinal sections in the resistant genotype at 14 dpi. RS, root surface; RV, root vascular bundle; HY, hypocotyl; ST, stem.

In the resistant *Luffa* genotype, LA140, the colonization of the pathogen in the roots, hypocotyl, and stem was monitored at four time points after infection (7, 14, 21, and 28 days). At 7 dpi, the heavy mycelium of the Fomh16-GFP isolate colonized the surface of the LA140 roots; at this time point, the mycelium had grown and spread throughout the root xylem vessels and cortex (Fig. 4A). Despite no symptoms appearing in resistant plants, the growth density of the mycelium was similar in the susceptible genotype at 7 dpi. After 14 dpi, there was a significant reduction in pathogen colonization on the root surface, and only the slightest colonization was observed in the root xylem (Fig. 4B). During this time, the resistant plant produced new and robust roots without any mycelia.

**Fig 5:**
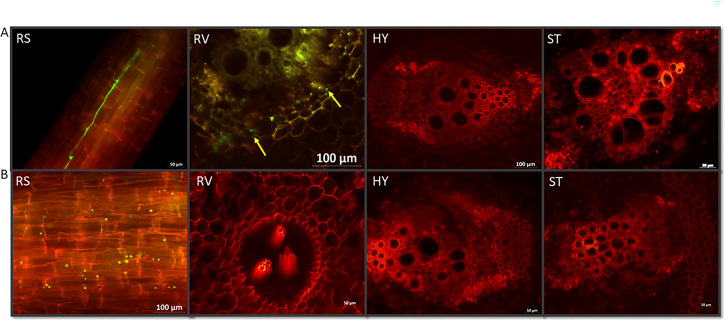
Infection and colonization of Folu Fomh16-GFP isolate in the resistant *Luffa* genotype LA140. (A) Cross and longitudinal sections in the resistant genotype at 21 dpi; (B) cross and longitudinal sections in the resistant genotype at 28 dpi. RS, root surface; RV, root vascular bundle; HY, hypocotyl; ST, stem.

After 21 dpi, the colonization of mycelium on the root surface showed a continued decrease. Furthermore, a small amount of mycelium invaded the root cortex cells, but none was found in the xylem vessels at this stage (Fig. 5A). By 28 dpi, only chlamydospores were observed to be attached to the root surface. However, almost all chlamydospores could not germinate (Fig. 5B). In addition, no mycelial growth was observed in the root vascular bundle, and the chlamydospores were only observed in small numbers on the oldest primary roots. In contrast, most secondary and tertiary roots remained healthy.

In the hypocotyl of LA140, at 7 and 14 dpi, slight mycelium had invaded and colonized the vascular bundle (Fig. 4). However, mycelium did not grow at 21 and 28 dpi in hypocotyl (Fig. 5). Additionally, no fungal structures, such as conidia and chlamydospores, were observed in hypocotyl tissues. In the stem, after analyzing over 1,200 stem sections of the resistant genotype LA140, it was observed that the mycelium of Fomh16-GFP was unable to infiltrate and colonize the stem vascular bundle or cortex until 28 dpi (Fig. 5B).

### Specificity of Fol02 and Fol03 primers to Folu

**Fig 6:**
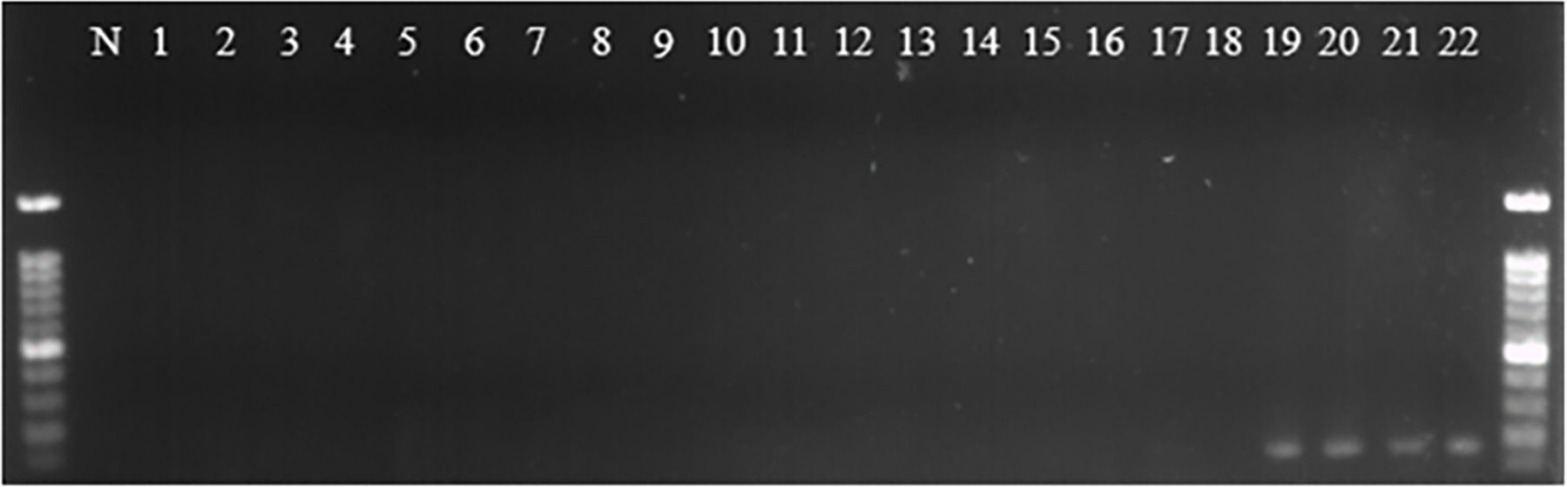
Specificity test of Fo102/Fo103 primer pairs using conventional polymerase chain reaction against 22 Fusarium isolates. Line N, negative control with sterile distilled water (SDW). Lines 1–16, *Fusarium oxysporum* isolates. Lines 17 and 18, *Fusarium solani* isolates. Lines 19 to 22, Folu isolates.

To successfully detect Folu using the qPCR assay, it is necessary to utilize efficient and specific primers. To verify primer specificity, we utilized the Fol02 and Fol03 primers to amplify the genomic DNA (gDNA) of 22 *Fusarium* spp. isolates, consisting of 20 *F*. *oxysporum* isolates and 2 *F. solani* isolates (Table 1), through conventional PCR. Based on the results, it was found that the primers Fol02 and Fol03 were able to only amplify a gDNA target fragment of 184 bp from *F. oxysporum* f. sp. *luffae* isolates FOLUST, FOLUSC, Fol114, and Fomh16-GFP (Fig. 6). There was no amplification of PCR products in the gDNA of other fungal isolates and non-template samples. This result suggests that the primers Fol02 and Fol03 are specific to only *F. oxysporum* f. sp. *luffae*. Real-time PCR was performed using these primers to measure the DNA quantity of the Folu isolate in both resistant and susceptible *Luffa* genotypes.

**TABLE 1:** Isolates of *Fusarium* species used in this study and the results of the specificity of the PCR primer pairs Fo102 and Fo103

No.	Isolate	*Fusarium* species	Host	Primer pairs Fo102/Fo103
1	Fomo33	*F. oxysporum* f. sp. *momordicae*	*Momordica charantia*	−
2	Fomo35	*F. oxysporum* f. sp. *momordicae*	*Momordica charantia*	−
3	Fon-K0105	*F. oxysporum* f. sp. *niveum*	*Citrullus lanatus*	−
4	Focl27	*F. oxysporum* f. sp. *cucumerinum*	*Cucumis sativus*	−
5	Fom3	*F. oxysporum* f. sp. *melonis*	*Cucumis melo*	−
6	Fola103-7	*F. oxysporum* f. sp. *lactucae*	*Lactuca sativa*	−
7	Fol 195	*F. oxysporum* f. sp. *lycopersici*	*Solanum lycopersicum*	−
8	Fol 146	*F. oxysporum* f. sp. *lycopersici*	*Solanum lycopersicum*	−
9	Focb-24	*F. oxysporum* f. sp. *cubense*	*Musa* sp.	−
10	Foli G16	*F. oxysporum* f. sp. *lilii*	*Lilium* sp.	−
11	FIA	*F. oxysporum* f. sp. *anoetochili*	*Anoectochilus formosanus*	−
12	F7C	*F. oxysporum* f. sp. *anoetochili*	*Anoectochilus formosanus*	−
13	St61	*F. oxysporum* f. sp. *anoetochili*	*Anoectochilus formosanus*	−
14	Eus5	*F. oxysporum*	*Eustoma grandiflorum*	−
15	CyB04	*F. oxysporum*	*Cymbidium ensifolium*	−
16	Cy32	*F. oxysporum*	*Cymbidium sinense*	−
17	Ph22	*F. solani*	*Phalaenopsis*	−
18	Ph64	*F. solani*	*Phalaenopsis*	−
19	FOLUST	*F. oxysporum* f. sp. *luffae*	*Luffa*	+
20	Fol114	*F. oxysporum* f. sp. *luffae*	*Luffa*	+
21	Fomh16	*F. oxysporum* f. sp. *luffae*	*Momordica charantia*	+
22	FOLUSC	*F. oxysporum* f. sp. *luffae*	*Luffa*	+

### Sensitivity of Fol02 and Fol03 primers for Folu and standard curve analysis

**Fig 7:**
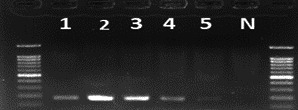
Sensitivity test of Fo102 and Fo103 primer pairs using conventional polymerase chain reaction with gDNA of Folu Fomh16-GFP isolate. Lines 1 to 5, Fomh16-GFP DNA concentration started from 25 ng/µL to 2.5 pg/µL; line N, negative control with SDW.

**Fig 8:**
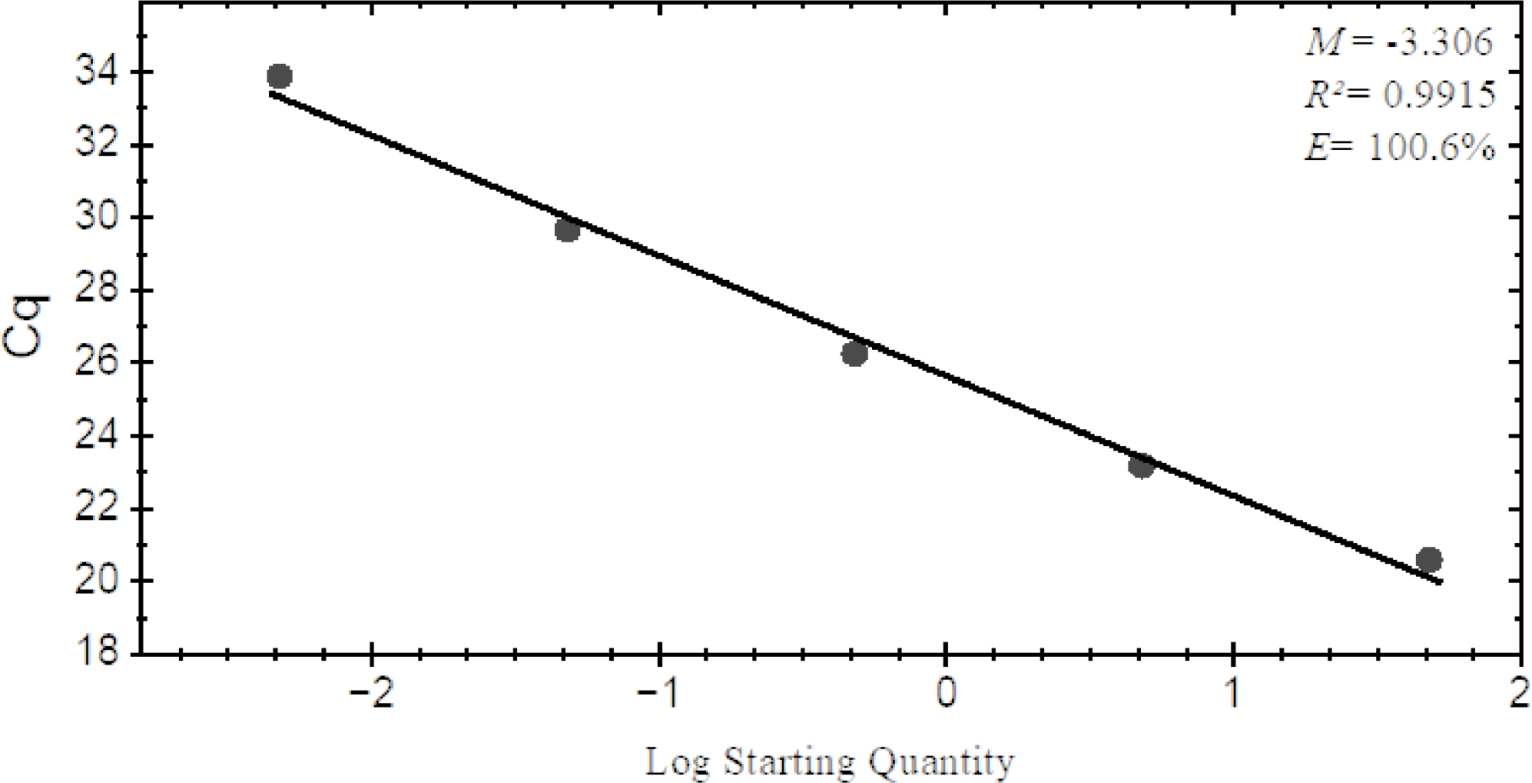
Real-time PCR assay standard curve of Folu Fomh16-GFP isolate using primer pairs Fo102/Fo103. Standard curve generated using a 10-fold serial dilution of Fomh16-GFP DNA ranging from 4.8 pg to 48 ng/µL. Each point represents the average of three biological replicates.

To test the sensitivity of the Fol02 and Fol03 primers, a 10-fold serial dilution ranging from 2.5 pg/µL to 25 ng/µL of *F. oxysporum* f. sp. *luffae* gDNA Fomh16-GFP isolate was used under PCR conditions. This experiment revealed that the Fol02 and Fol03 primers could amplify an 184-bp target fragment from as little as 2.5 pg/µL of Fomh16-GFP gDNA (Fig. 7). A standard curve was generated by performing 10-fold serial dilutions ranging from 4.8 pg/µL to 48 ng/µL of Fomh16-GFP gDNA. The real-time PCR results revealed that the primers Fol02 and Fol03 produced target peaks with a melting temperature of 82°C. The primers Fol02 and Fol03 achieved high efficiency in detecting DNA concentrations with the five different amounts of Fomh16-GFP gDNA studied, with a detection limit of a concentration of 4.8 pg/µL. A linear correlation was observed between the concentration of Fomh16-GFP gDNA and real-time quantification cycles (*R*^2^ = 0.9915) with high-efficiency rates (*E* > 100.6%) (Fig. 8).

### Detection and quantification of Folu in inoculated *Luffa* plants

**Fig 9:**
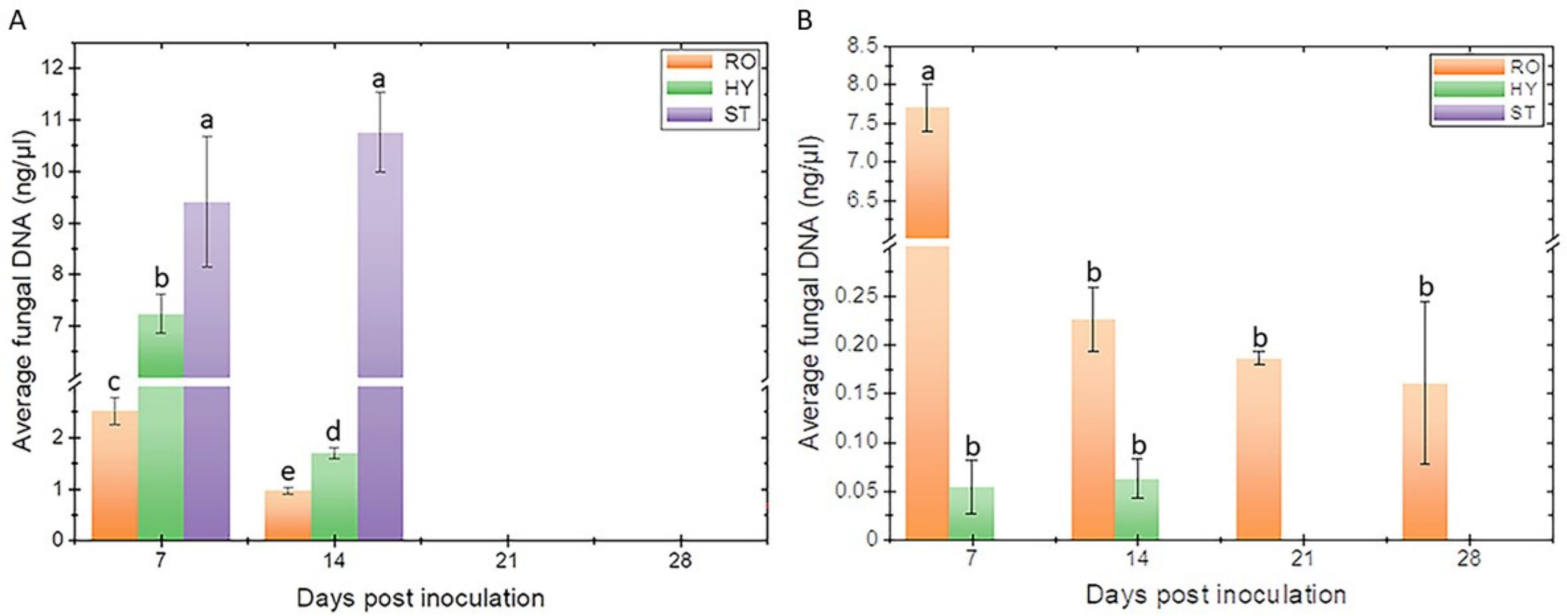
Quantity of Fomh16-GFP DNA in different tissues of *Luffa* plants at different time points after inoculation. (A) Susceptible genotype (LA100); (B) resistant genotype (LA140); RO, whole roots; HY, hypocotyl; ST, stem. In the case of A at 21 and 28 dpi, plant samples could not be collected because the plants were completely wilted by 2 weeks after infection. Different letters indicate significant differences between values according to Fisher’s protected least significant difference test (*P* = 0.05).

The real-time PCR results are shown in Fig. 9. In the roots of LA100, the high concentration of Fomh16-GFP DNA, measuring around 2.51 ng/µL of total plant DNA, was detected at 7 dpi. However, this amount decreased to 0.96 ng/µL of total plant DNA by day 14 (Fig. 9A). Similarly, in hypocotyl samples, the amount of Fomh16-GFP DNA was initially high at 7.23 ng/µL of total plant DNA but decreased to 1.69 ng/µL of total plant DNA by day 14. In the stem, a very high amount of Fomh16-GFP DNA, measuring 9.40 ng/µL and 10.69 ng/µL of total plant DNA, was detected at 7 and 14 dpi, respectively (Fig. 9A).

For the resistant genotype LA140, it was observed that at 7 dpi, the amount of pathogen DNA in the root samples was exceptionally high at 7.69 ng/µL of total plant DNA (Fig. 9B). However, interestingly, this amount was significantly reduced to 0.22 ng/µL of total plant DNA by 14 dpi. The concentration of pathogen DNA in the root samples continued to decrease over time, reaching 0.18 ng/µL and 0.16 ng/µL of the total plant DNA at 21 and 28 dpi, respectively. No significant differences were observed in the amount of Fomh16-GFP DNA in the hypocotyl samples of LA140. The measurements taken at 7 and 14 dpi showed that the amounts were 0.054 ng/µL and 0.062 ng/µL of total plant DNA, respectively. Throughout the experiment, none of the stem samples showed any detectable pathogen DNA (Fig. 9B).

### Antifungal activity of aqueous extract from resistant genotype

**Fig 10:**
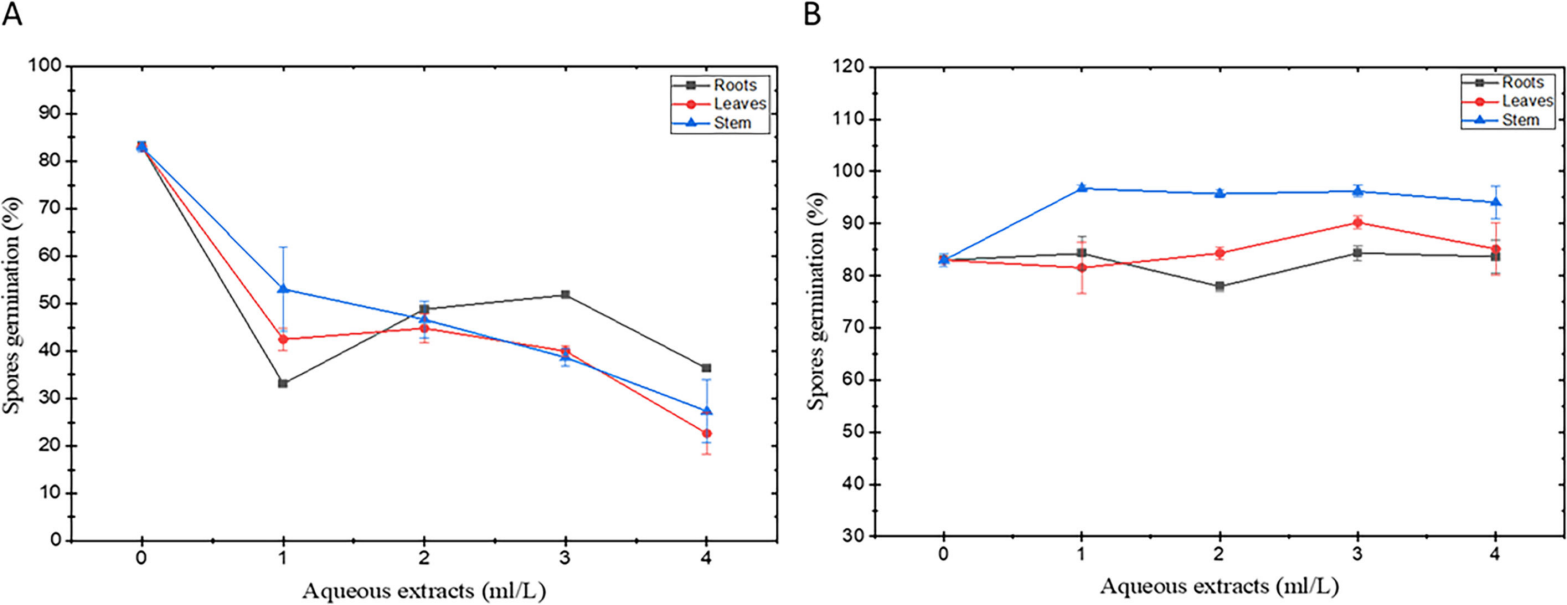
Effect of aqueous extracts of *Luffa* genotypes on spore germination of Folu Fomh16-GFP isolate. (A) Aqueous extracts from resistant genotype (LA140); (B) aqueous extracts from susceptible genotype (LA100).

According to the results, the aqueous extracts obtained from the roots, stem, and leaves of the resistant genotype have inhibitory effects on Fomh16-GFP spore germination at all concentrations. It was observed after 8 hr of incubation at 28°C and shown in Fig. 10. On the one hand, the leaf extract from LA140 resulted in the lowest spore germination percentage at 22.6%, stem extracts had a slightly higher rate at 27.3%, and root extracts had a percentage of 33.1%. On the other hand, the aqueous extracts from all parts of the susceptible genotype did not exhibit significant inhibitory effects compared with the control. Based on these results, it is evident that the aqueous extract of the resistant genotype LA140 showed a significant ability to inhibit Folu spore germination compared with the extracts obtained from the susceptible genotype LA100 (Fig. 10).

## DISCUSSION

In this study, fluorescence microscopy and real-time PCR were used to examine the colonization, distribution, and amount of *F. oxysporum* f. sp. *luffae* in both resistant and susceptible *Luffa* genotypes. Using the Fomh16-GFP, we examined the infection processes and colonization of Folu in *Luffa* genotypes. Moreover, there were no significant morphological and pathogenic differences between the Fomh16-GFP and wild-type isolates.

The disease progression in the susceptible genotype LA100 was monitored after infection with Fomh16-GFP. The results revealed that the pathogen could initially attach to the root surface at 0 to 7 dpi, penetrate the epidermal cell layer, and expand to colonize the cortex and xylem vessels of the roots, hypocotyl, and stem. These results showed similar phenomena of *F. oxysporum* f. sp. *niveum* in watermelon (19), f. sp. *fragariae* in strawberry (32), and f. sp. *cucumerinum* in cucumber (20, 33). Fomh16-GFP continually colonized both the root surface and vascular systems with high density, and the pathogen had almost completely colonized all plant parts in LA100 after 14 dpi. Similar results were reported in a previous study (34), where *F. oxysporum* f. sp. *ciceri* colonized all plant parts during disease progression in susceptible chickpea cultivars.

Moreover, the colonization dynamics of Fon in susceptible watermelon cultivars revealed a similar pattern: hyphae grew on the root epidermis within 2 dpi, penetrated the epidermal cells to reach the cortex by 3 dpi, and advanced into root xylem by 7 dpi (19). Thus, pathogenic *F. oxysporum* is capable of fully penetrating and colonizing the root surface and subsequently invading the cortex and xylem vessels of susceptible crops all by 6 to 7 dpi (5, 35). In tomato roots, the *F. oxysporum* f. sp. *radicis-lycopersici* colonizes the root surface and forms a network of hyphae. These hyphae expand and infect all junctions of the epidermal cells over time, leading to complete colonization of the root system (36, 37).

In the resistant genotype LA140, the colonization of the Fomh16-GFP isolate during the infection process was similar to that of the susceptible genotype LA100 at 7 dpi. Initially, the high-density mycelium colonized the root surface and subsequently penetrated the epidermal cell, extending its colonization to the cortex and xylem vessels of the roots. Previous studies reported that the initial infection stages of *F. oxysporum* in both resistant and susceptible watermelons and carnations were similar (35, 38). Moreover, Upasani et al. (34) indicated that the *F. oxysporum* f. sp. *ciceri* colonized the root surface of resistant or susceptible cultivars during the initial stages of infection; however, infection of the vascular system of resistant cultivars was slower and reaching the xylem vessels required more time than that in susceptible cultivars. Different results were presented by Cohen et al. (39), who noted that the mycelia of *F. oxysporum* f. sp. *radicis-cucumerinum* (Forc) in resistant melon cultivar colonized only a few intercellular spaces of the parenchyma, and none were detected in the vascular tissue. Thus, the colonization and penetration of pathogenic *F. oxysporum* to resistant lines or cultivars are associated with different crops. Therefore, the colonization pattern of Folu on resistant *Luffa* differs from that of Forc on resistant cucumber. It is necessary to determine the true resistance mechanism of *Luffa* to Folu in the future.

An interesting phenomenon was observed. Fomh16-GFP colonized the hypocotyl vascular system of the resistant genotype with a small amount of mycelia at 7 to 14 dpi. However, by 21 dpi, Fomh16-GFP could not be detected in any hypocotyl or root vascular tissue, and only very few mycelia were observed on the root surface, and throughout the experiment, no Fomh16-GFP mycelia were observed in the stem of LA140. These results suggested that the LA140 induced resistance after the pathogen had penetrated hypocotyl tissue. Zvirin et al. (40) indicated that in a resistant melon cultivar, Fom mycelia were restricted to the lower hypocotyl sections. Further research revealed that Fon colonized the vascular tissue of resistant watermelon but was then reduced a few days later (35). However, in some cases, it was shown that Fo *formae speciales* can colonize the stems of resistant and susceptible cultivars, such as watermelon (41) and tomato (42). In addition, whether pathogens could extend their colonization up to the stem was greatly influenced by the level of resistance exhibited by the plants (22).

Interestingly, by 28 dpi, the fungal colonization significantly decreased, only chlamydospores were found to adhere to the root surface, and no microconidia were observed in the resistant genotype. In contrast with the susceptible genotype, most of these residual chlamydospores in the resistant genotype could not germinate, with only a few producing very small mycelium. A previous study (23) reported that Fon could produce only a few secondary conidia on resistant watermelon roots. In this study, the variations in chlamydospore germination between susceptible and resistant genotypes may attributed to the root exudates from the resistant plants. Buxton (43) indicated that the root exudates of resistant plants significantly reduced the pathogenicity of *F. oxysporum* f. sp. *pisi* compared with susceptible plants. Similar findings were observed in *F. oxysporum* f. sp. *niveum* (44). However, different results were reported for *F. oxysporum* f. sp. *pisi* and *F. oxysporum* f. sp. *lycopersici*, where the host root exudates from susceptible or resistant cultivars could not affect the spore germination of two formae speciales (45, 46). The difference between our results and previous studies may be due to various factors that impact root exudates, including plant genotype, plant age, soil properties, and microorganisms (47–50).

It is important to note that even though the Fomh16-GFP isolates colonized the resistant genotype, no symptoms were exhibited until 28 dpi. These findings are similar to findings regarding *F. oxysporum* f. sp. *niveum* colonizing the roots of highly resistant watermelons and cultivars without displaying wilt symptoms (22, 24).

To better comprehend the correlation between Fomh16-GFP biomass and wilting symptoms, measuring the quantity of pathogen DNA in resistant and susceptible plants is crucial. Based on qPCR analysis, the Fomh16-GFP could be detected in the root, hypocotyl, and stem of the susceptible genotype (LA100) at 7 dpi with significantly high biomass. This result showed that within 7 dpi, the Fomh16-GFP easily penetrated and colonized the susceptible genotype. However, qPCR detection revealed that the Fomh16-GFP biomass decreased in the susceptible genotype’s roots and hypocotyl at 14 dpi. The DNA decrease in the susceptible plant may be attributed to the extensive wilting; as most root tissues decayed and these symptoms spread to the hypocotyl, the fungal biomass reduced. Previous studies have shown similar outcomes for Fon and Foc in susceptible watermelon (27) and chickpea (34), respectively. Thus, the pathogenic *F. oxysporum* could fast infect the root system of susceptible genotypes and cause plants to show root decay and wilting; however, when the plant tissue begins to disintegrate, the pathogens will decrease in plants but reside in debris.

On the other hand, Fomh16-GFP showed a much higher biomass in the resistant genotype (LA140) in roots at 7 dpi than the susceptible genotype. These results indicated that Fomh16-GFP could still grow and enclose on the root surface of the resistant genotype during the initial period after inoculation. However, Fomh16-GFP was significantly decreased in roots from 14 to 21 dpi. Zhong et al. (27) reported that resistant plants could induce a defense response after Fo penetration. Thus, Fomh16-GFP could grow on the root surface without penetration in the early stage; however, when Fomh16-GFP started to penetrate, the resistance of LA140 was induced to defend against Fomh16-GFP and caused the pathogen biomass to decrease. In this study, the pathogen could not be quantified in the stem sample during the experiment. This demonstrated that Fomh16-GFP was restricted to roots or hypocotyl in LA140. Several researchers indicated that non-pathogenic *F. oxysporum* or pathogenic *F. oxysporum* in resistant genotypes were restricted to only roots or hypocotyl and could not ascend to the stem (40, 51).

For antifungal activity tests, the crude aqueous extracts from resistant and susceptible genotype plants showed that the aqueous extracts from all resistant genotype parts (roots, stems, and leaves) significantly reduced Fomh16-GFP spore germination compared with the susceptible genotype. These results indicated that the crude aqueous extracts from the resistant genotype included antimicrobial compounds to inhibit spore germination and suggested that these antifungal compounds naturally existed in plants or were induced upon pathogen interaction ; however, more experiments are required. Moreover, whether or not these antifungal compounds could have systemic movement also needs to be analyzed in the future. Previous studies indicated that resistant crops could accumulate some secondary metabolites in xylem sap (15), including peroxidases and phenols that contribute to plant defense (52, 53). In certain *Luffa* species, more than 10 flavonoids, including luteolin-7-glucoside, have been detected in leaves and flowers (54). Báidez et al. (55) reported that the accumulation of luteolin-7-glucoside in the xylem of olive trees could be involved in the natural defense or resistance mechanism against *Verticillium dahlia*. Biles et al. (56) reported that the xylem exudates from cucumber, muskmelon, and tomato effectively inhibited Fon microconidia germination. More studies demonstrated that extracts from *L. acutangula* have efficacies on antimicrobial growth (57–59). Here, we extracted liquid from LA140 using ddH_2_O. However, this study did not use other organic solvents, such as methanol, ethanol, acetic acid, and chloroform (60, 61). Using different organic solvents might extract more antifungal compounds due to compound polarity. Different organic solvents will be used to extract more efficacious compounds in the future.

## MATERIALS AND METHODS

### Plant material and culture condition

The *Luffa* LA140 genotype belonging to *L. acutangula*, obtained from the GenBank of World Vegetable Center (WorldVeg), was used in this study. In our previous study, the LA140 genotype displayed high resistance to four Folu isolates (FOLUSC, FOLUST, Fol114, and Fomh16). Accession LA100, a commercial *Luffa* hybrid (cv. Shimmery), was obtained from Known-You Seed Co. Ltd. (Kaohsiung, Taiwan) and used as the susceptible control. The seeds were surface sterilized using 70% ethanol for 30 s and 1% NaOCl for 1 min. Afterwards, the seeds were rinsed three times with SDW to ensure consistent seed germination; the seeds were treated by cutting the seed coat opposite the embryo site, then soaked in SDW, and incubated at 30°C for 1 day. The seeds were sowed into plastic seedling trays containing an autoclaved peat moss and perlite soil mixture (3:1). The plants were grown in a greenhouse at 25 to 28°C, a relative humidity of 80%–85%, and a 12-hr photoperiod.

### *Agrobacterium tumefaciens*-mediated transformation of Fomh16

For carrying out the colonization and distribution of Fomh16 in *Luffa*, the Fomh16-GFP isolate was conducted. First, to determine the minimum inhibitory concentration of hygromycin B, wild-type isolates of Folu (Fomh16) were cultured on potato dextrose agar (PDA) with varying concentrations of hygromycin B including 50, 100, 200, 300, and 400 µg/mL. A total of 10 µL of fungus spore suspension adjusted to 1 × 10^6^ conidia mL^−1^ was spread on the PDA plates and incubated at 25°C for 14 days in the dark in triplicates. The *Agrobacterium tumefaciens* strain EHA105 containing the binary vector p1300-CT74 was kindly provided by Prof. Miin-Huey Lee (Department of Plant Pathology, National Chung Hsing University, Taiwan). The plasmid CT74 contains the hygromycin resistance gene (*hptII*) and the GFP under the *Aspergillus nidulans trp*C promoter (62). Folu Fomh16 isolate transformation was executed according to a method described previously with slight modifications (63). A single colony of the *A. tumefaciens* strain EHA105 was cultured in LB broth medium containing 50 µg/mL kanamycin and incubated at 28°C on a rotary shaker at 100 rpm overnight to reach OD_620_ = 0.5. Afterward, the *A. tumefaciens* cells were diluted to OD_620_ = 0.15 with induced minimal media broth supplemented with 40 mM MES hydrate and 200 mM acetosyringone. These cells were grown for an additional 4–6 hr at 28°C at 200 rpm. The conidia suspension (1.0 × 10^6^/mL) of Fomh16 isolate was prepared from a single spore culture grown on PDA media for 14 days mixed with an equal value of acetosyringone-induced *A. tumefaciens* cells (0.300 OD). A total of 200 µL of this mix was spread on a 47-mm diameter cellulose nitrate membrane filter with 0.45-µm pores (Whatman, Japan) that covered the co-cultivation medium (64) supplemented with 200 µM acetosyringone. Plates were incubated at 25°C in the dark for 3 days, and then, the membranes were moved into PDA selective media containing 200 µg/mL hygromycin B and 200 µM cefotaxime sodium salt to eliminate the *A. tumefaciens* cells. These plates were incubated for an additional 7 days at 25°C in the dark. The individual transformant fungal colonies were transferred five times to PDA selective media to ensure the stability of transformation and morphology. Then, the morphology and pathogenicity were examined to confirm the Fomh16-GFP showing similar characteristics to the wild-type isolate.

### Inoculum preparation and inoculation

A GFP-tagged isolate (Fomh16-GFP) of *F. oxysporum* f. sp. *luffae* was used to inoculate the susceptible LA100 and resistant LA140 *Luffa* genotypes. Plants were inoculated using a root-dipping method described previously (16). In brief, conidial suspensions were prepared from single-spore cultures grown on PDA and incubated for 10 to 14 days at 28°C. The concentration of conidial suspension was adjusted to 2.5 × 10^6^ conidia mL^−1^. Healthy plants with one to two true leaves were uprooted. Roots were washed under a stream of gently flowing water, and a third of the root tips was trimmed off and submerged in the Fomh16-GFP conidial suspension for 30 min. Control plants were dipped in SDW after trimming. The inoculated and control seedlings were maintained in a greenhouse at 22°C–28°C and 83%–86% humidity. The seedlings were watered daily and fertilized weekly with fertilizer containing nitrogen, phosphorus, and potassium (20%–20%–20%). The plants were arranged in a randomized complete block design, with three replicates per accession, eight for each replicate, and eight uninoculated plants were used as mock controls.

The plants were independently evaluated every 7 days up to 28 dpi. The disease severity was assessed based on a 0–5 rating scale, where 0 = no apparent disease symptoms, 1 = yellowing in the cotyledon leaves, 2 = yellowing or wilting of one true leaf, 3 = either the wilting of two true leaves or plant stunting, 4 = wilting of a majority of plant leaves, and 5 = plant death. The MDR was calculated at 28 dpi according to the following formula: MDR = Σ(N*_i_* × *i*)/N*_t_*, where N*_i_* indicates the number of plants that received each disease rating score *i* and N*_t_* indicates the total number of plants. *Luffa* genotypes were classified based on their resistance to the Folu Fomh16-GFP isolate using the MDR results. Genotypes with MDR {less than or equal to} 1, 1 < MDR {less than or equal to} 2, 2 < MDR {less than or equal to} 3, and MDR > 3" were considered resistant, moderately resistant, moderately susceptible, and susceptible, respectively.

### Molecular identification of the transformed Fomh16-GFP isolate

To verify the existence of the *hptII* and GFP genes in the transformed isolate, the mycelia from 10- to 14-day-old cultures of the transformed (Fomh16-GFP) and wild-type (Fomh16) isolates were scraped from the PDA plates. gDNA was extracted using a DNeasy Blood & Tissue Kit (QIAGEN GmbH, Hilden, Germany). PCR was performed using a PCR Master Mix (BioKit, Taiwan); the 25-µL PCR mixture contained 0.5 µL (10 µmol/L) of each primer and 1 µL of DNA template (25 ng/reaction). The two specific primers HygR3 (5′-GGATGCCTCCGCTCGAAGTA-3′) and HygR5 (5′-CTTAAGTTCGCCCTTCCTCC-3′) and GFP_F (5′-ATGGTGAGCAAGGGCGAGGA-3′) and GFP_R (5′-TTACTTGTACAGCTCGTCCA-3′) were used to amplify the *hptII* and GFP genes, respectively, in the transformed and wild-type isolates. A non-template DNA sample was used as a negative control. The PCR conditions were set in Labcycler 48 (SensoQuest GmbH, Göttingen, Germany) as follows: denaturation at 94°C for 5 min followed by 35 cycles of 95 for 30 s and annealing for 30 s at 55°C, then 1 min at 72°C, and followed by 10 min at 72°C for extension. The PCR products were subjected 1.5% agarose gel electrophoresis (PC Biotech, Taiwan). The PCR products were visualized and photographed under UV light.

### Microscopic observations

The resistant LA140 and susceptible LA100 *Luffa* genotypes were inoculated with Fomh16-GFP isolate. The inoculated and control plants were sampled weekly for 4 weeks. Six plants from each genotype were selected and gently rinsed with tap water during each sampling period to remove soil particles. Cross or longitudinal sections were prepared using an electro-freeze machine MA-101 (Komatsu Electronics, Japan). These sections were placed directly on glass slides in drops of SDW and covered with a glass coverslip for microscopy observation. The colonization of the pathogen on the entire root surface and vascular system of roots, hypocotyl (1 cm above the soil), and stem (2 cm above cotyledon leaves) were observed using an Axio Imager A1 microscope (Carl Zeiss AG, Jena, Germany) equipped with a fluorescence illuminator system X-Cite 120Q (Excelitas Technologies, USA) and Axiocam 506 color camera. To visualize the fluorescence expression of the Fomh16-GFP isolate, an optical filter (CHROMA 31001) with excitation at 480/12 nm and emission at 535/30 nm was used. Plant autofluorescence was detected using an optical filter (CHROMA 31,002a) with excitation at 540/25 nm and emission at 620/60 nm. The experiment was performed in triplicate and repeated three times, and about 30 slices were observed per replication in each plant part.

### Primer design, specificity, and sensitivity tests

Two specific primers, Fol02 (5′-TCCAGACAAACGCGCTATTC-3′) and Fol03 (5′-CTTCACCACAATAACGCCGA-3′), were designed to investigate the distribution and quantification of Folu Fomh16-GFP isolate in the roots, hypocotyls, and stems of LA100 and LA140 *Luffa* genotypes. Folu*-*specific primer sequences, fp7320 and fp7337 (65), were used for primer design using Primer3Plus software (https://www.bioinformatics.nl/cgi-bin/primer3plus/primer3plus.cgi). The specificity of the Fol02 and Fol03 primers was evaluated against 22 isolates of *Fusarium* spp., including 20 isolates of *F. oxysporum* and two isolates of *F. solani*; these isolates were collected from different regions of Taiwan (Table 1). The Fusarium isolates were grown on PDA media for 10–14 days at 28°C, and gDNA extraction followed a method described previously (16). The 25-µL PCR mixture contained 0.5 µL (10 µmol/L) of each primer and 1 µL of DNA template (25 ng/reaction). PCR conditions were set in a programmed temperature control system (Astec Co. Ltd., Tokyo, Japan) as follows: denaturation at 94°C for 2 min followed by 35 cycles of denaturation for 30 s at the same temperature, annealing for 30 s at 60°C, then extension for 40 s at 72°C, followed by 5 min at 72°C, and a pause at 16°C. All experiments included the gDNA of the *F. oxysporum* f. sp. *luffae* Fomh16-GFP isolate and sterile Milli-Q water as positive and negative controls, respectively, to detect any DNA contamination of reagents and reaction mixtures. The PCR assay was analyzed using a 1.5% agarose gel containing FluoroStain DNA Fluorescent Staining Dye (SMOBIO Technology Inc., Taiwan) at 0.2 mg/mL.

To detect the primers Fol02 and Fol03 with high sensitivity, a 10-fold serial dilution of gDNA of the Fomh16-GFP isolate was performed, starting from 2.5 pg/µL to 25 ng/µL. The 25 µL PCR mixture contained 1.0 µL of each dilution as a DNA template and 0.5 µL (10 µmol/L) of each primer. The gDNA extraction, PCR conditions, and gel electrophoresis were used as described above. The standard curve was generated using 10-fold serial dilutions (1:1, 1:10, 1:10^2^, 1:10^3^, and 1:10^4^) of Fomh16-GFP gDNA 48 ng/µL in SDW, and the concentration was adjusted using an EzDrop 1000 (Blue-Ray Biotech, Taiwan). The qPCR conditions were similar to those described below. These samples were amplified in three biological replicates, and the quantified target DNA and Cq values of the serial dilution performed the standard curves.

### Distribution and quantification of Fomh16-GFP *in planta*

The distribution and the amount of Fomh16-GFP DNA in different parts of LA100 and LA140 *Luffa* genotypes were estimated using qPCR. Inoculated *Luffa* plants were collected at 7, 14, 21, and 28 dpi. Whole plant roots were ground in liquid nitrogen to ensure a homogenous sample. Next, DNA was extracted from 200 mg of the root, hypocotyl, and stem using a Plant Genomic DNA Purification Kit from Genemark Technology Co. Ltd. in Taiwan. This DNA was then used as the template for further analysis. A qPCR analysis was conducted using a CFX 384 Real-Time PCR System CFX 384 (Bio-Rad Laboratories, California, USA). Each 10 µL of PCR reaction contained 5 µL of PowerUp SYBR Green Master Mix (Applied Biosystems, USA), 0.5 µL (10 µmol/L) of each primer, 3 µL of RNase-free ddH_2_O, and 1 µL of DNA template. The DNA concentration was adjusted to 25 ng/µL before use; non-template samples and DNA from uninoculated plants were used as negative controls. The samples were amplified in triplicate, and a standard curve was run in each experiment. The thermocycling condition consisted as follows: 50°C for 2 min, denaturation at 95°C for 2 min, followed by 40 cycles of denaturation at 95°C for 15 sec, annealing at 60°C for 1 min. The concentration of Fomh16-GFP DNA in different plant parts of the inoculated *Luffa* genotypes was estimated by comparing them with the standard curve.

### Plant aqueous extracts and spore germination assay

A previous study indicated one resistance mechanism might be plants producing chemicals to reduce the colonization or penetration of pathogens (56). Here, aqueous extracts of resistant LA140 were extracted to test whether antifungal compounds were produced by resistant LA140. Aqueous extracts were prepared by collecting plant samples (roots, stems, and leaves) from susceptible and resistant genotypes. The plant tissues were first ground in liquid nitrogen and soaked in SDW at a 1:10 ratio (wt/vol) and incubated at a temperature of 28°C with 200 rpm for 24 hr. Next, the mixtures were filtered using two layers of cheesecloth and centrifuged for 4 min at 8,000 × *g* to remove any particulate material. These extracts were sterilized using a 0.45-µm syringe filter and stored in the dark at a temperature of −20°C. To investigate the impact of crude aqueous extract on Fomh16-GFP spore germination, the spore suspension was adjusted to a concentration of 1.0 × 10^6^/mL. Various concentrations (1, 2, 3, and 4 mL/L) of aqueous extract were mixed with the spore suspension, and 10 µL was placed on a cavity slide and incubated at 28°C. After 8 hr of incubation, the spore germination percentage was observed and recorded under a microscope.

### Statistical analysis

Data analyses were conducted using the R Statistical Software v4.1.3 (https://www.r-project.org/). To analyze the statistical significance of the *Luffa* genotypes on mean disease rating, we used non-parametric Kruskal-Wallis analysis and Dunn tests for multiple comparisons (*P* < 0.05). One-way analysis of variance and Fisher’s least significant difference multiple comparisons test at *P* < 0.05 were used to analyze the significance of the amount of pathogen in *Luffa* genotypes.
